# Mandibular Cortical Thickness Predicts Skull BMD in Adolescents

**DOI:** 10.1007/s00223-026-01501-1

**Published:** 2026-03-06

**Authors:** Tarsila de Moura Figueiredo, Vid Prijatelj, Olja Grgic-Chavez, Carolina Medina-Gomez, Eppo Wolvius, Lea Kragt, Fernando Rivadeneira

**Affiliations:** 1 Department of Oral and Maxillofacial Surgery, Special Dental Care and Orthodontics, Erasmus MC, University Medical Center Rotterdam, Doctor Molewaterplein 40, 3015 GD Rotterdam, The Netherlands; 2 The Generation R Study Group, Erasmus MC, University Medical Center Rotterdam, Doctor Molewaterplein 40, 3015 GD Rotterdam, The Netherlands; 3 Department of Internal Medicine, Erasmus MC, University Medical Center Rotterdam, Doctor Molewaterplein 40, 3015 GD Rotterdam, The Netherlands

**Keywords:** Panoramic radiography, Polygenic score, Bone mineral density, Biomarkers, Mandible, Puberty

## Abstract

Bone mass accrual during childhood and early adulthood is the most important modifiable determinant of lifelong skeletal health. Dental panoramic radiographs (DPRs) and measurements derived from them have yet to be assessed as a screening tool for low bone mineral density in children. Skull bone mineral density (SK-BMD) has high heritability and is less affected by environmental factors than measures of BMD at other skeletal sites. This study aimed to analyze the relationship between mandibular cortical thickness and SK-BMD in peripubertal children. This population-based cross-sectional study involved 2672 children aged 13 years from the Generation R Study. Mandibular cortical thickness was obtained from DPRs and quantified with the mental index (MI) and the superior panoramic mandibular index (sPMI). SK-BMD was quantified from total body dual-energy X-ray absorptiometry scans and weighted polygenic scores (wPGS). The determinants of mandibular cortical thickness were also assessed. The analysis was done with linear regression models, adjusted for confounders, and within a Mendelian randomization (MR) framework. Both MI and sPMI were significantly associated with SK-BMD in fully adjusted models. When the characteristics of the participants stratified for the extreme quintiles of the SK-BMD wPGS distribution were compared, only SK-BMD (*p* value < 0.001), sPMI (*p* value = 0.032), and MI (*p* value < 0.001) differed significantly between extremes. Mandibular cortical thickness parameters were significantly associated with SK-BMD in an unconfounded setting. DPRs may serve as a valuable tool for surmising low BMD in peripubertal children, once clinical cutoffs for mandibular cortical thickness are established.

**Supplementary Information:**

The online version contains supplementary material available at 10.1007/s00223-026-01501-1.

## Introduction

Bone mineral density (BMD) is the amount of mineralized bone content per square centimeter in a given region of interest [1]. Dual-energy X-ray absorptiometry (DXA) is considered the gold standard to measure BMD [2]. Osteoporosis is characterized by low BMD, which increases fracture risk and diminishes the quality of life [3, 4]. Peak bone mass is an important determinant of BMD in the life course as it refers to the state of maximum accretion of skeletal mass. Environmental factors may be modified in childhood to maximize BMD before peak bone mass is reached. Approximately 26% of the final adult total body bone mass is accrued during the 2-years surrounding the period of peak height velocity [5]. By 18 years of age, 90% of peak bone mass is already achieved [1]. Therefore, bone mass accrual during childhood and early adulthood is an important modifiable determinant of lifelong skeletal health.

In children, skeletal development is assessed through DXA scans using total body less head BMD. However, DXA requires high initial and operational costs which restrict its availability [6]. Hence, radiographs have been proposed as a possible alternative to DXA scans [7]. Dental panoramic radiographs (DPRs) have been proposed as a useful tool for assessing low BMD in adults [8]. To that end, many quantitative indices have been proposed. Within those, the mandibular cortical thickness has been reported as having the best specificity for low BMD [9]. Previously, we showed that mandibular cortical thickness and total body less-head BMD are associated and may share underlying biological pathways [10]. Since the head is excluded from BMD evaluation in pediatric populations, it is unclear if DPRs parameters correlate with SK-BMD [11].

SK-BMD has been shown to have weaker associations with anthropometric, body composition, and lifestyle factors than other skeletal sites. Interestingly, despite less mechanical loading, calvarial osteocytes have been shown to maintain bone mass [12]. As such, SK-BMD has emerged as a relevant parameter for bone research. It has high heritability and is considerably less affected by environmental influences [13]. Due to those unique characteristics, SK-BMD captures more efficiently the genetic architecture of clinically relevant skeletal sites [14]. Analyzing SK-BMD thus provides an opportunity to better elucidate an individual’s genetic potential for bone mass accrual while serving the purpose of standing as a proxy of the gold standard for BMD evaluation in children.

Previous genome-wide association studies (GWAS) have identified genetic variants influencing SK-BMD and demonstrated that, while SK-BMD is genetically correlated to BMD at other skeletal sites, it also has specific loci, such as variants mapping to *EYA4* and *LIN7C* [14, 15]. Our study employs a design similar to that used by “Recall-by-Genotype” (RbG) studies [16]. RbG studies seek recalling participants from the extremes of a genotype distribution for further phenotyping with the advantage of drawing comparisons in an “unconfounded” setting. This is possible due to the Mendelian randomization (MR) framework underlying a given polygenic score (PGS) distribution, where the random allocation of alleles (at conception) results in the randomization of confounding factors across PGS subgroups. Using this approach in an observational population-based study, allows us to evaluate the association between mandibular cortical thickness and SK-BMD in an unconfounded setting [17].

Given the high accessibility of DPRs in clinical dental practice, we aimed to assess their potential use for the identification of low BMD in children. Thus, the aim of this study is to analyze the relationship between mandibular cortical thickness and skull bone mineral density in healthy peripubertal children. The secondary objective is to describe the determinants of mandibular cortical thickness. The association established in an observational setting is further assessed using the MR framework.

## Materials and Methods

### Study Design

#### Study Population

This cross-sectional study is embedded in the Generation R Study [18]. Pregnant women who resided in Rotterdam, the Netherlands, within 2002–2006 were recruited. The children were assessed periodically via repeated questionnaires and measurements during visits to the research center. At approximately 13 years old, 4929 children had their growth assessed. Of those, 2672 participants had complete information on mandibular cortical thickness, SK-BMD and other relevant measurements. This study was approved by the Medical Ethics Committee of the Erasmus Medical Center, Rotterdam (MEC-2015-749), and followed the guidelines of the Helsinki Declaration. Study participants provided written informed consent at each phase of the study, and from the age of 12, children signed their own consent forms in accordance with Dutch Law. This cross-sectional study follows the STROBE (Strengthening the Reporting of Observational Studies in Epidemiology) reporting guidelines.

#### SK-BMD Assessment

SK-BMD was measured directly and by proxy using a PGS. Total body DXA scans were performed to obtain direct measurements (GE-Lunar iDXA scanner, GE Healthcare, Madison, WI, USA) according to manufacturer instructions. Qualified research assistants obtained the measurements. A daily quality control protocol was followed similarly to previous visits. Before scanning, participants were asked to remove shoes, heavy clothing, metallic accessories, and anything else that could affect the procedure [19]. The skull region of interest was extracted from the DXA scans. SK-BMD (g/cm^2^) was calculated using the enCORE software (v13.60).

#### Genotyping and SK-BMD PGS Calculation

DNA samples from cord blood or blood samples collected at the visit at 6 years old were genotyped in two phases, using Illumina HumanHap 610 or 660 Quad chips (Illumina Inc., CA, USA), and Global Screening Array-Molecular Diagnostics GSA-MD (GSA-MD) v2 arrays (Illumina Inc., CA, USA). Quality control, phasing, and imputation procedures for both phases have been described in detail previously [20, 21]. The PGS was created using summary statistics from a GWAS on SK-BMD [15]. Palindromic variants as well as variants with low minor allele frequency (MAF < 0.01) and insufficient imputation quality (Rsq < 0.8) were excluded. Information was obtained for 79 independent SNPs that were found to be genome-wide significant (*p* value < 5 × 10^−8^) after performing conditional and joint multiple-SNP analysis [15]. Dosage information from the same variants was extracted from imputed data of participants. Data was harmonized so that each extracted variant allele was trait-increasing. A weighted PGS (wPGS) was calculated wherein allele dosages were multiplied by their respective effect size and the products summed together [22].

#### Mandibular Cortical Thickness Assessment

The mandibular cortical thickness was based on DPRs, obtained from an orthopantomograph OP200D device (Oldelft Benelux B.V., Veenendaal, the Netherlands). Prior to scanning, participants removed any accessories that could present themselves as artifacts in the radiographs. Chin rests or bite blocks were provided to those missing front teeth or having malocclusion. Scans were taken following manufacturer specifications. The mandibular cortical thickness was quantified by two individual measures: MI and sPMI. Based on the DPRs, a trained dentist calculated both indices for each individual. A subset of 150 radiographs were re-evaluated and evaluated by a second dentist for inter- and intraclass correlation analyses. MI expresses the cortical thickness on an axis that crosses the approximate center of the mental foramen and forms a 90 angle with an axis tangential to the lower mandibular rim. sPMI is the ratio of MI and the distance between the mental foramen’s upper border and the mandible’s lower border on the same vertical axis [8]. These measurements were taken bilaterally, and the arithmetic mean was calculated. When both mental foramina were not clearly visible, the sinistral side was used since it was more often available. All measures were performed using the Viewbox 4 software (dHAL Software, Kifissia, Greece).

#### Migration Background

Migration background was established at enrollment based on the country of birth of the parents according to the Statistics Netherlands (CBS) classification and has been described elsewhere [23]. The diverse migration backgrounds were combined in three main categories: Europeans (all European countries, Americans, Oceanics, and North Africans), Asians (all Asian countries and Surinamese Hindustanis), and Africans (sub-Saharan Africans, Dutch Antilleans, and Surinamese Creoles) [24].

#### Ancestral Background

Genetically determined ancestral background was utilized to identify participants of European descent for inclusion in the MR framework. Genetic ancestry for each individual was established using ADMIXTURE [25]. Based on population allele frequency and the highest fraction of the estimated ancestral proportion (> 50%), individuals were clustered into three main groups: African (AFR), European (EUR), and Asian (ASN). Participants were classified as “admixed” if none of the calculated ancestral proportions exceeded 50% [26].

#### Puberty Scores

A questionnaire including seven questions regarding growth spurt, body hair growth, facial hair growth for boys, skin changes related to acne, voice changes for boys, breast development for girls and menarche for girls was filled in by the participants. The answers were used to calculate a pubertal score. All questions, except for the one on menarche, had five possible answers and were graded as follows: not yet started (1 point), barely started (2 points), definitively started (3 points), seems complete (4 points), and not known (missing). Menarche was a dichotomous outcome graded as: yes (4 points) and no (1 point). The point values were averaged for all items and summed to give the puberty score [27]. This questionnaire has been validated and shows sufficient accuracy to distinguish prepubertal and pubertal stages [28].

#### Additional Covariates

Sex and date of birth were obtained from the medical records and hospital registries. Height was measured using a Harpenden stadiometer (Holtain Limited, Dyfed, U.K.). Weight was determined with a mechanical personal scale (SECA, Almere, the Netherlands). BMI was calculated as body weight (kg)/height (m)^2^.

### Statistical Analysis

The baseline descriptive characteristics of study participants were assessed using T-tests for continuous variables and chi-squared tests for categorical variables and presented stratified by sex. The association between mandibular cortical thickness and SK-BMD was explored using ordinary-least squares linear regression. Prior to running the model, these parameters were standardized using Z-score (normal) transformation for ease of interpretation. The standardized variables were then labeled as sPMI_Z, MI_Z, and SK-BMD_Z. Using multivariable models, the analysis was adjusted for confounders in a stepwise fashion as follows. Model 0 had only the raw association. Model 1 included age, sex, BMI, height and migration background. Model 2 contained the aforementioned covariates and puberty score. European migration background and male sex were defined as reference categories.

The effect of each determinant on mandibular cortical thickness was analyzed with univariate linear regression models. The marginal effect was assessed in two multivariable models. Model 1 contained all covariates except puberty and Model 2 included all covariates. Sensitivity analyses were performed with participants of European migration background and with missing puberty scores imputed using imputation by chained equations with 10 imputations. The baseline characteristics of participants with and without puberty information were compared with Student's *t*-tests, Mann–Whitney *U* tests or Chi-square tests.

The MR framework was used to mitigate the impact of unmeasured confounding. It was performed in participants of European ancestral background. Study participants were allocated along the wPGS distribution. Before comparing the extremes of the distribution, a power calculation to define extremes’ cutoffs was performed [17]. The parameters were sample size, number of quantiles, R^2^ between PGS and exposure as reported before [15], R^2^ between exposure and outcome, and alpha level. The analysis indicated sufficient statistical power to define quintiles as cutoff points. Individuals were stratified into quintiles based on their wPGS. The relevance assumption of the instrumental variable was assessed by regressing SK-BMD on the wPGS. The F-statistic was used to evaluate instrumental variable strength. Subsequently, each covariate was regressed on the wPGS to investigate the independence assumption. Characteristics of the participants in the bottom (1st) and top (5th) quintiles were compared using a T-test or a Chi-squared test. All statistical analyses were performed with R statistical software, version 4.1.2 (R Foundation for Statistical Computing).

## Results

### Population Characteristics

The observational analysis included 2672 children (1268 boys; 47.5%) with median age of 13.5 (IQR = 0.3) years old (Table 1). A total of 2254 children were of European migration background (84.4%). The mean sPMI and MI were 0.28 and 6.21 mm, respectively. The mean SK-BMD was 1.70 g/cm^2^. Girls had significantly higher measurements of SK-BMD, mandibular cortical thickness, BMI and puberty score than boys (*p* value < 0.001).

**Table 1:** Population characteristics of the study sample stratified by sex

	Boys	Girls	Total	*p* value
N*	1268 (47.5)	1404 (52.5)	2672	
SK-BMD (g/cm^2^)	1.63 (0.17)	1.77 (0.21)	1.70 (0.20)	**< 0.001**
sPMI	0.27 (0.05)	0.29 (0.05)	0.28 (0.05)	**< 0.001**
MI (mm)	6.12 (1.19)	6.29 (1.02)	6.21 (1.10)	**< 0.001**
Age (Years)	13.61 (0.34)	13.60 (0.36)	13.60 (0.35)	0.461
Weight (kg)	53.32 (10.96)	54.31 (10.52)	53.84 (10.74)	0.017
Height (cm)	165.75 (8.78)	163.94 (6.91)	164.80 (7.90)	**< 0.001**
BMI (kg/m^2^)	19.29 (3.01)	20.15 (3.44)	19.74 (3.27)	**< 0.001**
Migration background*				
African	142 (11.2)	139 (9.9)	281 (10.5)	0.456
Asian	68 (5.4)	69 (4.9)	137 (5.1)	
European	1058 (83.4)	1196 (85.2)	2254 (84.4)	
Puberty score	2.04 (0.63)	2.82 (0.61)	2.45 (0.74)	**< 0.001**

Values are expressed as means with their corresponding standard deviation. Significant *p* values are represented in bold

Significance threshold ≤ 0.001

*N* sample size, *SK-BMD* skull bone mineral density, *sPMI* superior panoramic mandibular index, *MI* mental index, *BMI* body mass index

*Count percentage

### Mandibular Cortical Thickness and Skull Bone Mineral Density

In the raw model, each standard deviation (SD) increase of sPMI was associated with a 0.22 SD increase of SK-BMD (Table 2) (*p* value < 2 × 10^−16^). Each SD increase of MI was associated with a 0.34 SD increase of SK-BMD (*p* value < 2 × 10^−16^). In model 1, each SD increase of sPMI and MI was associated with a 0.12 and 0.25 SD increase of SK-BMD, respectively (*p* value = 6.7 × 10^−12^, *p* value < 2 × 10^−16^). In model 2, the effect sizes of the sPMI and MI associations were slightly decreased and remained significant (respectively β = 0.11 and 0.23, *p* value = 6.01 × 10^−10^ and < 2 × 10^−16^). Sensitivity analyses on children of European background only (Table S1) and with imputed puberty status (Table S2) followed the original models’ trends. Participants with puberty score information were more often of European migration background and 0.39 cm taller than those who had this information missing (*p* value < 0.05).

**Table 2:** Association between mandibular cortical thickness and SK-BMD measured in children aged 13 years

	Boys (N = 1268)						Girls (N = 1404)						Total (N = 2672)					
	Model 0	Model 1	Model 2	Model 0	Model 1	Model 2	Model 0	Model 1	Model 2	Model 0	Model 1	Model 2	Model 0	Model 1	Model 2	Model 0	Model 1	Model 2
sPMI_Z	**0.11 (0.023)**	**0.08 (0.024)**	**0.09 (0.024)**				**0.23 (0.027)**	**0.18 (0.026)**	**0.15 (0.025)**				**0.22 (0.019)**	**0.12 (0.018)**	**0.11 (0.018)**			
MI_Z				**0.23 (0.021)**	**0.20 (0.023)**	**0.21 (0.023)**				**0.43 (0.027)**	**0.34 (0.026)**	**0.31 (0.026)**				**0.34 (0.018)**	**0.25 (0.018)**	**0.23 (0.018)**
Age (Years)		0.17 (0.071)	0.17 (0.071)		0.13 (0.069)	0.13 (0.069)		**0.40 (0.068)**	**0.34 (0.066)**		**0.40 (0.066)**	**0.35 (0.064)**		**0.29 (0.050)**	**0.27 (0.049)**		**0.27 (0.048)**	**0.25 (0.048)**
Sex (Girl)														**0.66 (0.035)**	**0.49 (0.043)**		**0.67 (0.034)**	**0.53 (0.042)**
BMI (kg/m^2^)		**0.03 (0.008)**	**0.03 (0.008)**		0.02 (0.008)	0.02 (0.008)		**0.07 (0.007)**	**0.04 (0.008)**		**0.05 (0.007)**	**0.03 (0.007)**		**0.055 (0.005)**	**0.046 (0.006)**		**0.042 (0.005)**	**0.036 (0.005)**
Height (cm)		0.01 (0.003)	**0.01 (0.003)**		0.003 (0.003)	0.01 (0.003)		**0.03 (0.004)**	**0.02 (0.004)**		**0.03 (0.003)**	**0.02 (0.004)**		0.017 (0.002)	0.0097 (0.002)		0.011 (0.002)	0.0059 (0.002)
Migration background (African)		0.08 (0.075)	0.09 (0.075)		0.02 (0.073)	0.03 (0.073)		0.13 (0.082)	0.07 (0.079)		0.08 (0.079)	0.03 (0.076)		0.105 (0.056)	0.081 (0.056)		0.049 (0.055)	0.033 (0.055)
Migration background (Asian)		− 0.18 (0.103)	− 0.15 (0.104)		− 0.21 (0.101)	− 0.17 (0.102)		0.20 (0.113)	0.06 (0.110)		0.15 (0.109)	0.04 (0.106)		− 0.039 (0.077)	**− 0.101 (0.077)**		− 0.078 (0.076)	**− 0.12 (0.076)**
Puberty score			− 0.10 (0.045)			**− 0.15 (0.044)**			**0.46 (0.044)**			**0.42 (0.043)**			0.21 (0.032)			0.17 (0.032)

Results are presented for each sex and for the whole study population separately. Table represents linear regression coefficients and standard erros

Significance threshold ≤ 0.001. Significant estimates are represented in bold

Model 0: raw association. Model 1: sPMI_Z or MI_Z and age, sex, BMI, height and migration background. Model 2: sPMI_Z or MI_Z and age, sex, BMI, height, migration background and puberty score

*sPMI* superior panoramic mandibular index, *MI* mental index, *BMI* body mass index. *Z* standardized values

### Determinants of Mandibular Cortical Thickness

In the univariate models sex, BMI, African migration background and puberty score were significantly associated with sPMI (respectively β = 0.33, 0.065, 0.35 and 0.29) (Table S3). All covariates, besides Asian migration background (*p* value = 0.67), had a significant effect on MI. In model 1, the relationship between the determinants and sPMI or MI resembled that observed in univariate models with the exception of age (*p* value > 0.001). In model 2, BMI (β = 0.047, *p* value = 1.1 × 10^−14^), African migration background (β = 0.27, *p* value = 1.3 × 10^−5^), and puberty score (β = 0.19, *p* value = 3.4 × 10^−8^) were significantly associated with sPMI. The aforementioned parameters and height (β = 0.013, *p* value = 8.6 × 10^−7^) were significantly and positively associated with MI. The effect of sex on sPMI and MI was attenuated and no longer significant once the puberty score was included.

### MR Framework

**Fig. 1:**
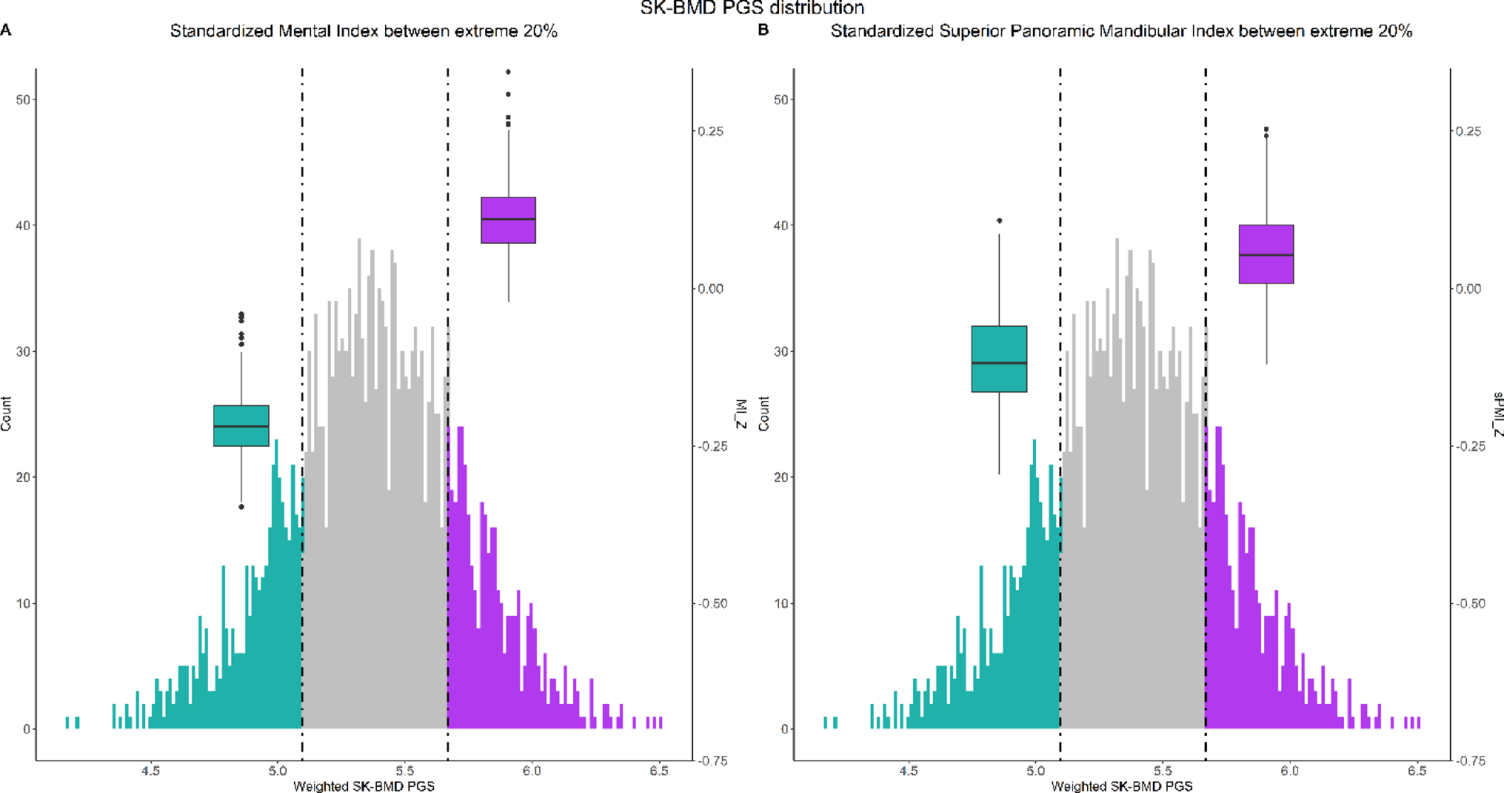
Distribution of the skull bone mineral density (SK-BMD) weighted polygenic score (wPGS). Dashed lines mark and colored areas highlight the 20% extreme quintiles. Boxplots show the distribution of mandibular cortical thickness indices in children in the extreme quintiles of the PGS distribution. **A** Standardized superior panoramic index (sPMI_Z). **B** Mental index (MI_Z)

A total of 2118 participants of European ancestral background had genetic data available for PGS calculation. The wPGS was a strong instrumental variable for SK-BMD (β = 0.8, *p* value < 2 × 10^−16^, F statistic = 183.8). It was not associated with age (*p* value = 0.3, F statistic = 1.1), BMI (*p* value = 0.4, F statistic = 0.7), sex (*p* value = 0.6), and puberty (*p* value = 0.2, F statistic = 1.9), ensuring the wPGS was independent of confounding factors. According to the power calculation, the study was sufficiently powered (1 − β > 0.8) to compare individuals grouped in the 1st and 5th quintiles of the distribution. SK-BMD (*p* value < 0.001), sPMI (*p* value = 0.032) (Fig. 1), and MI (*p* value < 0.001) were significantly higher in the top quintile compared to the bottom quintile, while other parameters were randomized (Fig. 1 and Table 3).

**Table 3:** Representation of skull bone mineral density (SK-BMD), mandibular cortical thickness indices and other covariates for children at the 20% extreme quintiles of the SK-BMD polygenic score (PGS) distribution

	Bottom 20%	Top 20%	*p* value
Stratified by quintile			
N	424	424	
SK-BMD_Z (mean (SD))	− 0.40 (0.90)	0.42 (0.95)	**< 0.001**
sPMI_Z (mean (SD))	− 0.09 (0.97)	0.05 (0.98)	**0.032**
MI_Z (mean (SD))	− 0.21 (0.88)	0.13 (0.96)	**< 0.001**
Age (Years) (mean (SD))	13.62 (0.35)	13.58 (0.33)	0.132
BMI (mean (SD))	19.60 (3.05)	19.55 (2.76)	0.791
Sex (Girl)*	219 (51.7)	235 (55.4)	0.302
Height (mean (SD))	164.82 (7.68)	165.16 (7.75)	0.526
Puberty score (mean (SD))	2.45 (0.75)	2.40 (0.76)	0.281

Significance threshold ≤ 0.05. Significant estimates are represented in bold

*N* sample size, *SD* standard deviation, *SK-BMD* skull bone mineral density, *sPMI* superior panoramic mandibular index, *MI* mental index, *BMI* body mass index

*Count percentage

## Discussion

This report demonstrates the association between mandibular cortical thickness (sPMI and MI) and SK-BMD in a healthy peripubertal population. These findings were confirmed in an unconfounded setting, by accounting for various confounders in the model and by using an MR framework underlying the PGS distribution. Individuals in the lower quintile of the SK-BMD wPGS distribution had lower measurements for SK-BMD, sPMI and MI.

The clinical relevance of SK-BMD is two-fold. First, it captures the individual genetic potential for skeletal development more effectively than BMD measurements at weight-bearing skeletal sites, which are more influenced by environmental factors [14, 29]. Second, SK-BMD is strongly correlated with BMD at clinically relevant sites, such as the lumbar spine [15]. Thus, individuals predisposed to low peak bone mass may be identified through DPR examination. Clinical guidelines for this approach and clinical cutoffs for mandibular cortical thickness have been established for adults [30]. However, evaluating bone mass in childhood and adolescence provides an opportunity to intervene and reduce the elevated risk of a clinically significant fracture in the future [31]. Early intervention could be more beneficial, with adequate nutrition and weight-bearing exercise showing the strongest evidence of benefit [32].

The MR framework confirmed the unconfounded association between SK-BMD and mandibular cortical thickness. SK-BMD, sPMI and MI were significantly different between quintiles, unlike other measured parameters. These findings may be explained by a shared biological background. Genetic variants linked to SK-BMD may influence craniofacial development. For example, *EN1* gene expression is associated with femoral cortical thickness, SK-BMD and craniosynostosis [33]. Additionally, a *SMAD9* mutation reduces bone morphogenetic protein (BMP) inhibition, increasing osteoblast activity and results in mandible enlargement [34]. Future studies should explore the genetic mechanisms underlying this association.

Several individual characteristics significantly affected mandibular cortical thickness. Children of sub-Saharan African background had higher sPMI and MI, which is consistent with a previous study reporting greater cortical thickness at several skeletal sites in African–American young adults [35]. Genetic variation related to migration background partly explains these differences [36]. Importantly, correction for migration background did not affect the association between mandibular cortical thickness and skull BMD, making our findings generalizable to children from diverse backgrounds. BMI, which is associated with higher BMD in weight-bearing sites, was positively associated with sPMI and MI, suggesting a systemic effect on bone metabolism [37]. This association has been previously reported for overweight and obese children [38]. Previous studies have shown that boys have significantly thicker mandibular cortices than girls, but that such sexual dimorphism can be masked by pubertal status [39, 40]. In this study, no differences in mandibular cortical thickness were observed between sexes after puberty score adjustment. As such, migration background, BMI and pubertal status must be considered during DPR assessment.

DPRs provide a broad view of the maxillomandibular area, making them the preferred method for general oral health assessment. In children, they are recommended during the transitional dentition phase, after the eruption of the first permanent tooth, and before the eruption of the third molars [41]. Therefore, DPRs are a frequently used diagnostic tool in pediatric dental evaluations. DPRs and other dental radiographs are so widely used that they represent 30% of all radiographic examinations. However, due to the low exposure to ionizing radiation, they contribute only 4% of the total collective effective radiation dose [42]. In addition, DPRs also have low cost compared to other imaging techniques and intraoral radiographs [43]. Besides dental and craniofacial conditions, we have demonstrated that DPRs can be used to detect low BMD in children. However, DPRs should not replace DXA scan evaluations but serve as an adjunct evaluation tool to identify high-risk children undergoing dental assessments who might otherwise remain undetected.

A limitation of this study was the underrepresentation of other migration backgrounds, as the sample consisted mostly of European children. Future studies should investigate other confounders, such as physical activity or diet. The MR framework was ancestral background specific to avoid population stratification issues. Since the GWAS results were derived from a sample of mostly European individuals (85%), our analyses included only those of European ancestry. Additionally, DPR use has disadvantages such as distortion of three-dimensional structures and lower resolution, compared to other dental radiograph modalities [44]. Moreover, clinical cutoffs for low mandibular cortical thickness in children have yet to be established. However, given their accessibility, we aimed to contribute to validating DPRs as an evaluation tool.

Here we described an association between SK-BMD and mandibular cortical thickness as well as the determinants of mandibular cortical thickness in healthy children. A notable strength of this study was the application of a MR framework to analyze the significant association of these traits within an observational context. We suggest that DPRs may serve as a tool for identifying children with low BMD.

## Supplementary Information

Below is the link to the electronic supplementary material.

Supplementary Material 1

## References

[1] Gordon CM, Zemel BS, Wren TA, Leonard MB, Bachrach LK, Rauch F, Gilsanz V, Rosen CJ, Winer KK (2017) The determinants of peak bone mass. J Pediatr 180:261–269 27816219 10.1016/j.jpeds.2016.09.056

[2] Guss CE, McAllister A, Gordon CM (2021) DXA in children and adolescents. J Clin Densitom 24:28–35 32111573 10.1016/j.jocd.2020.01.006

[3] Gao S, Zhao Y (2023) Quality of life in postmenopausal women with osteoporosis: a systematic review and meta-analysis. Qual Life Res 32:1551–1565 36383282 10.1007/s11136-022-03281-1

[4] Hu J, Zheng W, Zhao D, Sun L, Zhou B, Liu J, Wang O, Jiang Y, Xia W, Xing X, Li M (2021) Health-related quality of life in men with osteoporosis: a systematic review and meta-analysis. Endocrine 74:270–280 34165773 10.1007/s12020-021-02792-0

[5] Bailey DA, McKay HA, Mirwald RL, Crocker PR, Faulkner RA (1999) A six-year longitudinal study of the relationship of physical activity to bone mineral accrual in growing children: the university of Saskatchewan bone mineral accrual study. J Bone Miner Res 14:1672–1679 10491214 10.1359/jbmr.1999.14.10.1672

[6] Sim MFV, Stone MD, Phillips CJ, Cheung WY, Johansen A, Vasishta S, Pettit RJ, Evans WD (2005) Cost effectiveness analysis of using quantitative ultrasound as a selective pre-screen for bone densitometry. Technol Health Care 13:75–85 15912005

[7] Shalof H, Dimitri P, Shuweihdi F, Offiah AC (2021) Which skeletal imaging modality is best for assessing bone health in children and young adults compared to DXA? A systematic review and meta-analysis. Bone 150:116013 34029779 10.1016/j.bone.2021.116013

[8] Mansour S, AlGhamdi AS, Javed F, Marzouk H, Khan EA (2013) Panoramic radiomorphometric indices as reliable parameters in predicting osteoporosis. Am J Med Sci 346:473–478 23811576 10.1097/MAJ.0b013e3182972148

[9] Calciolari E, Donos N, Park JC, Petrie A, Mardas N (2015) Panoramic measures for oral bone mass in detecting osteoporosis: a systematic review and meta-analysis. J Dent Res 94:17S-27S 25365969 10.1177/0022034514554949 PMC4541087

[10] Prijatelj V, Grgic-Chavez O, van der Tas J, Andaur Navarro CL, Uitterlinden AG, Rivadeneira F, Wolvius EB, Medina-Gomez C (2025) Genetic and epidemiologic assessment of mandibular cortical indices and bone mineral density in peripubertal children: the generation R study. medRxiv. 10.1101/2025.05.16.25327746v1 10.1007/s00784-025-06653-2 PMC12644194 41284120

[11] Crabtree NJ, Arabi A, Bachrach LK, Fewtrell M, El-Hajj Fuleihan G, Kecskemethy HH, Jaworski M, Gordon CM, International Society for Clinical D (2014) Dual-energy X-ray absorptiometry interpretation and reporting in children and adolescents: the revised 2013 ISCD pediatric official positions. J Clin Densitom 17:225–242 24690232 10.1016/j.jocd.2014.01.003

[12] Vatsa A, Breuls RG, Semeins CM, Salmon PL, Smit TH, Klein-Nulend J (2008) Osteocyte morphology in fibula and calvaria—Is there a role for mechanosensing? Bone 43:452–458 18625577 10.1016/j.bone.2008.01.030

[13] Moon RJ, D’Angelo S, Crozier SR, Godfrey KM, Davies JH, Cooper C, Harvey NC (2022) Is the skull responsive to bone mineralisation stimuli in children? Bone 160:116415 35398588 10.1016/j.bone.2022.116415

[14] Kemp JP, Medina-Gomez C, Estrada K, St Pourcain B, Heppe DH, Warrington NM, Oei L, Ring SM, Kruithof CJ, Timpson NJ, Wolber LE, Reppe S, Gautvik K, Grundberg E, Ge B, van der Eerden B, van de Peppel J, Hibbs MA, Ackert-Bicknell CL, Choi K, Koller DL, Econs MJ, Williams FM, Foroud T, Zillikens MC, Ohlsson C, Hofman A, Uitterlinden AG, Davey Smith G, Jaddoe VW, Tobias JH, Rivadeneira F, Evans DM (2014) Phenotypic dissection of bone mineral density reveals skeletal site specificity and facilitates the identification of novel loci in the genetic regulation of bone mass attainment. PLoS Genet 10:e1004423 24945404 10.1371/journal.pgen.1004423 PMC4063697

[15] Medina-Gomez C, Mullin BH, Chesi A, Prijatelj V, Kemp JP, Shochat-Carvalho C, Trajanoska K, Wang C, Joro R, Evans TE, Schraut KE, Li-Gao R, Ahluwalia TS, Zillikens MC, Zhu K, Mook-Kanamori DO, Evans DS, Nethander M, Knol MJ, Thorleifsson G, Prokic I, Zemel B, Broer L, McGuigan FE, van Schoor NM, Reppe S, Pawlak MA, Ralston SH, van der Velde N, Lorentzon M, Stefansson K, Adams HHH, Wilson SG, Ikram MA, Walsh JP, Lakka TA, Gautvik KM, Wilson JF, Orwoll ES, van Duijn CM, Bonnelykke K, Uitterlinden AG, Styrkarsdottir U, Akesson KE, Spector TD, Tobias JH, Ohlsson C, Felix JF, Bisgaard H, Grant SFA, Richards JB, Evans DM, van der Eerden B, van de Peppel J, Ackert-Bicknell C, Karasik D, Kague E, Rivadeneira F (2023) Bone mineral density loci specific to the skull portray potential pleiotropic effects on craniosynostosis. Commun Biol 6:691 37402774 10.1038/s42003-023-04869-0 PMC10319806

[16] Corbin LA-O, Tan VY, Hughes DA, Wade KH, Paul DA-O, Tansey KE, Butcher F, Dudbridge F, Howson JM, Jallow MW, John C, Kingston N, Lindgren CM, O’Donavan MA-O, O’Rahilly SA-O, Owen MA-O, Palmer CA-O, Pearson ER, Scott RA, van Heel DA-O, Whittaker J, Frayling T, Tobin MA-O, Wain LA-O, Smith GD, Evans DM, Karpe F, McCarthy MA-O, Danesh J, Franks PW, Timpson NJ (2018) Formalising recall by genotype as an efficient approach to detailed phenotyping and causal inference. Nat Commun 9:711 29459775 10.1038/s41467-018-03109-y PMC5818506

[17] Corbin LJ, Tan VY, Hughes DA, Wade KH, Paul DS, Tansey KE, Butcher F, Dudbridge F, Howson JM, Jallow MW, John C, Kingston N, Lindgren CM, O’Donavan M, O’Rahilly S, Owen MJ, Palmer CNA, Pearson ER, Scott RA, van Heel DA, Whittaker J, Frayling T, Tobin MD, Wain LV, Smith GD, Evans DM, Karpe F, McCarthy MI, Danesh J, Franks PW, Timpson NJ (2018) Formalising recall by genotype as an efficient approach to detailed phenotyping and causal inference. Nat Commun 9:711 29459775 10.1038/s41467-018-03109-y PMC5818506

[18] Kooijman MN, Kruithof CJ, van Duijn CM, Duijts L, Franco OH, van IMH, de Jongste JC, Klaver CC, van der Lugt A, Mackenbach JP, Moll HA, Peeters RP, Raat H, Rings EH, Rivadeneira F, van der Schroeff MP, Steegers EA, Tiemeier H, Uitterlinden AG, Verhulst FC, Wolvius E, Felix JF, Jaddoe VW (2016) The Generation R study: design and cohort update 2017. Eur J Epidemiol 31:1243–1264 28070760 10.1007/s10654-016-0224-9 PMC5233749

[19] Heppe DHM, Taal HR, Ernst GDS, Van Den Akker ELT, Lequin MMH, Hokken-Koelega ACS, Geelhoed JJM, Jaddoe VWV (2012) Bone age assessment by dual-energy X-ray absorptiometry in children: An alternative for X-ray? Br J Radiol 85:114–120 21586503 10.1259/bjr/23858213 PMC3473959

[20] Medina-Gomez C, Felix JF, Estrada K, Peters MJ, Herrera L, Kruithof CJ, Duijts L, Hofman A, van Duijn CM, Uitterlinden AG, Jaddoe VW, Rivadeneira F (2015) Challenges in conducting genome-wide association studies in highly admixed multi-ethnic populations: the generation R study. Eur J Epidemiol 30:317–330 25762173 10.1007/s10654-015-9998-4 PMC4385148

[21] Prijatelj V, Grgic O, Uitterlinden AG, Wolvius EB, Rivadeneira F, Medina-Gomez C (2024) Bone health index in the assessment of bone health: the generation R study. Bone 182:117070 38460828 10.1016/j.bone.2024.117070

[22] Choi SW, Mak TS, O’Reilly PF (2020) Tutorial: a guide to performing polygenic risk score analyses. Nat Protoc 15:2759–2772 32709988 10.1038/s41596-020-0353-1 PMC7612115

[23] Alders M (2001) Classification of the population with a foreign background in the Netherlands. In: Statistic Netherlands, paper for the conference “The measure and mismeasure of populations. The statistical use of ethnic and racial categories in multicultural societies”, Paris, p 18

[24] Medina-Gomez C, Heppe DHM, Yin JL, Trajanoska K, Uitterlinden AG, Beck TJ, Jaddoe VWV, Rivadeneira F (2016) Bone mass and strength in school-age children exhibit sexual dimorphism related to differences in lean mass: the generation R study. J Bone Miner Res 31:1099–1106 26599073 10.1002/jbmr.2755

[25] Alexander DH, Novembre J, Lange K (2009) Fast model-based estimation of ancestry in unrelated individuals. Genome Res 19:1655–1664 19648217 10.1101/gr.094052.109 PMC2752134

[26] Medina-Gomez C, Chesi A, Heppe DH, Zemel BS, Yin JL, Kalkwarf HJ, Hofman A, Lappe JM, Kelly A, Kayser M, Oberfield SE, Gilsanz V, Uitterlinden AG, Shepherd JA, Jaddoe VW, Grant SF, Lao O, Rivadeneira F (2015) BMD loci contribute to ethnic and developmental differences in skeletal fragility across populations: assessment of evolutionary selection pressures. Mol Biol Evol 32:2961–2972 26226985 10.1093/molbev/msv170 PMC4651235

[27] Carskadon MA, Acebo C (1993) A self-administered rating scale for pubertal development. J Adolesc Health 14:190–195 8323929 10.1016/1054-139x(93)90004-9

[28] Rasmussen AR, Wohlfahrt-Veje C, Tefre de Renzy-Martin K, Hagen CP, Tinggaard J, Mouritsen A, Mieritz MG, Main KM (2015) Validity of self-assessment of pubertal maturation. Pediatrics 135:86–93 25535262 10.1542/peds.2014-0793

[29] Nishi K, Endo D, Hasegawa T, Moriuchi T, Ogami-Takamura K, Saiki K, Murai K, Higashi T, Tsurumoto T, Manabe Y (2022) Similarities and differences in bone mineral density between multiple sites in the same individual: an elderly cadaveric study. Biomed Res Int 2022:6094663 35711524 10.1155/2022/6094663 PMC9197619

[30] Taguchi A, Tanaka R, Kakimoto N, Morimoto Y, Arai Y, Hayashi T, Kurabayashi T, Katsumata A, Asaumi J, Japanese Society for Oral and Maxillofacial Radiology (2021) Clinical guidelines for the application of panoramic radiographs in screening for osteoporosis. Oral Radiol 37:189–208 33620644 10.1007/s11282-021-00518-6

[31] Shuhart CR, Yeap SS, Anderson PA, Jankowski LG, Lewiecki EM, Morse LR, Rosen HN, Weber DR, Zemel BS, Shepherd JA (2019) Executive summary of the 2019 ISCD position development conference on monitoring treatment, DXA cross-calibration and least significant change, spinal cord injury, peri-prosthetic and orthopedic bone health, transgender medicine, and pediatrics. J Clin Densitom 22:453–471 31400968 10.1016/j.jocd.2019.07.001

[32] Weaver CM, Gordon CM, Janz KF, Kalkwarf HJ, Lappe JM, Lewis R, O’Karma M, Wallace TC, Zemel BS (2016) The National Osteoporosis Foundation’s position statement on peak bone mass development and lifestyle factors: a systematic review and implementation recommendations. Osteoporos Int 27:1281–1386 26856587 10.1007/s00198-015-3440-3 PMC4791473

[33] Kague E, Medina-Gomez C, Boyadjiev SA, Rivadeneira F (2022) The genetic overlap between osteoporosis and craniosynostosis. Front Endocrinol (Lausanne) 13:1020821 36225206 10.3389/fendo.2022.1020821 PMC9548872

[34] Gregson CL, Bergen DJM, Leo P, Sessions RB, Wheeler L, Hartley A, Youlten S, Croucher PI, McInerney-Leo AM, Fraser W, Tang JC, Anderson L, Marshall M, Sergot L, Paternoster L, Davey Smith G, Consortium A, Brown MA, Hammond C, Kemp JP, Tobias JH, Duncan EL (2020) A rare mutation in *SMAD9* associated with high bone mass identifies the SMAD-dependent BMP signaling pathway as a potential anabolic target for osteoporosis. J Bone Miner Res 35:92–105 31525280 10.1002/jbmr.3875 PMC7004081

[35] Popp KL, Hughes JM, Martinez-Betancourt A, Scott M, Turkington V, Caksa S, Guerriere KI, Ackerman KE, Xu C, Unnikrishnan G, Reifman J, Bouxsein ML (2017) Bone mass, microarchitecture and strength are influenced by race/ethnicity in young adult men and women. Bone 103:200–208 28712877 10.1016/j.bone.2017.07.014

[36] Grgic O, Shevroja E, Dhamo B, Uitterlinden AG, Wolvius EB, Rivadeneira F, Medina-Gomez C (2020) Skeletal maturation in relation to ethnic background in children of school age: the generation R study. Bone 132:115180 31786375 10.1016/j.bone.2019.115180

[37] Song J, Zhang R, Lv L, Liang J, Wang W, Liu R, Dang X (2020) The relationship between body mass index and bone mineral density: a Mendelian randomization study. Calcif Tissue Int 107:440–445 32989491 10.1007/s00223-020-00736-w

[38] Yasa Y, Buyuk SK, Genc E (2020) Comparison of mandibular cortical bone among obese, overweight, and normal weight adolescents using panoramic mandibular index and mental index. Clin Oral Investig 24:2919–2924 31802243 10.1007/s00784-019-03158-7

[39] Fan Y, Penington A, Kilpatrick N, Hardiman R, Schneider P, Clement J, Claes P, Matthews H (2019) Quantification of mandibular sexual dimorphism during adolescence. J Anat 234:709–717 30834524 10.1111/joa.12949 PMC6481415

[40] Maki K, Miller A, Okano T, Shibasaki Y (2000) Changes in cortical bone mineralization in the developing mandible: a three-dimensional quantitative computed tomography study. J Bone Miner Res 15:700–709 10780862 10.1359/jbmr.2000.15.4.700

[41] Menaker NH, Yepes JF, Vinson LA, Jones JE, Downey T, Tang Q, Maupomé G (2022) Prescription of bite-wing and panoramic radiographs in pediatric dental patients: an assessment of current trends and provider compliance. J Am Dent Assoc 153:23–30 34654530 10.1016/j.adaj.2021.07.001

[42] Kühnisch J, Anttonen V, Duggal MS, Spyridonos ML, Rajasekharan S, Sobczak M, Stratigaki E, Van Acker JWG, Aps JKM, Horner K (2020) Best clinical practice guidance for prescribing dental radiographs in children and adolescents: an EAPD policy document. Eur Arch Paediatr Dent 21:375–386 31768893 10.1007/s40368-019-00493-x

[43] Tsiklakis K, Mitsea A, Tsichlaki A, Pandis N (2020) A systematic review of relative indications and contra-indications for prescribing panoramic radiographs in dental paediatric patients. Eur Arch Paediatr Dent 21:387–406 31602555 10.1007/s40368-019-00478-w

[44] Peretz B, Gotler M, Kaffe I (2012) Common errors in digital panoramic radiographs of patients with mixed dentition and patients with permanent dentition. Int J Dent 2012:584138 22505905 10.1155/2012/584138 PMC3296161

