# Supplementary material for: Mandibular Cortical Thickness Predicts Skull BMD in Adolescents
Source: Calcif Tissue Int. 2026 Mar 6;117(1):36. doi: 10.1007/s00223-026-01501-1 (PMC12963067; doi:10.1007/s00223-026-01501-1)
Supplement: Supplementary file 1 — Supplementary Material 1 [file 223_2026_1501_MOESM1_ESM.docx]

***SUPPLEMENTARY TABLES***

**Table S1.** Association between mandibular cortical thickness and SK-BMD in a subset of 2254 children of European migration background only. Table represents linear regression coefficients and standard erros.

| **SK-BMD_Z** | | | | | | |
| --- | --- | --- | --- | --- | --- | --- |
|  | Model 0 | Model 1 | Model 2 | Model 0 | Model 1 | Model 2 |
| sPMI_Z | **0.21 (0.020)** | **0.11 (0.019)** | **0.098 (0.019)** |  |  |  |
| MI_Z |  |  |  | **0.34 (0.020)** | **0.23 (0.019)** | **0.22 (0.019)** |
| Years (Age) |  | **0.30 (0.053)** | **0.28 (0.053)** |  | **0.28 (0.052)** | **0.27 (0.052)** |
| Sex (Girl) |  | **0.64 (0.037)** | **0.48 (0.046)** |  | **0.63 (0.036)** | **0.505 (0.045)** |
| BMI (kg/m2) |  | **0.058 (0.006)** | **0.048 (0.006)** |  | **0.046 (0.006)** | **0.039 (0.006)** |
| Height (cm) |  | **0.017 (0.002)** | **0.0095 (0.003)** |  | **0.012 (0.002)** | **0.0064 (0.003)** |
| Puberty score |  |  | **0.205 (0.034)** |  |  | **0.16 (0.034)** |

sPMI = superior panoramic mandibular index, MI = mental index, BMI = body mass index. Z = standardized values. Significance threshold ≤ 0.001. Significant estimates are bolded. Model 0: raw association. Model 1: sPMI_Z or MI_Z and age, sex, BMI, and height. Model 2: sPMI_Z or MI_Z and age, sex, BMI, height, and puberty score.

**Table S2.** Association between mandibular cortical thickness and SK-BMD within 3542 participants with 870 participants having their puberty score imputed. Table represents linear regression coefficients and standard erros.

|  | **sPMI_Z** | | | **MI_Z** | | |
| --- | --- | --- | --- | --- | --- | --- |
|  | Model 0 | Model 1 | Model 2 | Model 0 | Model 1 | Model 2 |
| sPMI_Z | **0.23 (0.016)** | **0.13 (0.015)** | **0.12 (0.015)** |  |  |  |
| MI_Z |  |  |  | **0.35 (0.016)** | **0.25 (0.015)** | **0.23 (0.016)** |
| Age (Years) |  | **0.28 (0.039)** | **0.26 (0.039)** |  | **0.25 (0.038)** | **0.23 (0.038)** |
| Sex (Girl) |  | **0.69 (0.030)** | **0.52 (0.039)** |  | **0.69 (0.029)** | **0.56 (0.038)** |
| BMI (kg/m2) |  | **0.048 (0.004)** | **0.040 (0.004)** |  | **0.037 (0.004)** | **0.031 (0.004)** |
| Height (cm) |  | **0.019 (0.002)** | **0.012 (0.002)** |  | **0.013 (0.002)** | **0.0078 (0.002)** |
| Migration background (African) |  | 0.092 (0.045) | 0.071 (0.045) |  | 0.038 (0.044) | 0.023 (0.044) |
| Migration background (Asian) |  | -0.0098 (0.062) | -0.066 (0.063) |  | -0.041 (0.061) | -0.085 (0.061) |
| Puberty score |  |  | **0.20 (0.030)** |  |  | **0.16 (0.030)** |

sPMI = superior panoramic mandibular index, MI = mental index, BMI = body mass index. Z = standardized values. Significance threshold ≤ 0.001. Significant estimates are bolded. Model 0: raw association. Model 1: sPMI_Z or MI_Z and age, sex, BMI, height and migration background. Model 2: sPMI_Z or MI_Z and age, sex, BMI, height, migration background and puberty score.

**Table S3.** Determinants of mandibular cortical thickness assessed for 2672 participants. Table represents linear regression coefficients.

|  | **Model 0** | | **Model 1** | | **Model 2** | |
| --- | --- | --- | --- | --- | --- | --- |
|  | MI_Z | sPMI_Z | MI_Z | sPMI_Z | MI_Z | sPMI_Z |
| Age (Years) | **0.32 (0.055)** | 0.15 (0.055) | 0.12 (0.053) | 0.081 (0.054) | 0.094 (0.052) | 0.061 (0.054) |
| Sex (Girl) | **0.16 (0.039)** | **0.33 (0.038)** | **0.13 (0.037)** | **0.29 (0.047)** | -0.082 (0.045) | 0.13 (0.047) |
| BMI (kg/m2) | **0.093 (0.006)** | **0.065 (0.006)** | **0.078 (0.006)** | **0.056 (0.006)** | **0.066 (0.006)** | **0.047 (0.006)** |
| Height (cm) | **0.025 (0.002)** | 0.000073 (0.002) | **0.022 (0.002)** | -0.00097 (0.003) | **0.013 (0.003)** | -0.0075 (0.003) |
| Migration background (African) | **0.42 (0.063)** | **0.35 (0.063)** | **0.37 (0.059)** | **0.29 (0.061)** | **0.34 (0.059)** | **0.27 (0.061)** |
| Migration background (Asian) | 0.18 (0.087) | 0.037 (0.087) | 0.15 (0.082) | -0.0043 (0.085) | 0.075 (0.082) | -0.061 (0.085) |
| Puberty score | **0.39 (0.025)** | **0.29 (0.026)** |  |  | **0.27 (0.034)** | **0.19 (0.035)** |

sPMI = superior panoramic mandibular index, MI = mental index, BMI = body mass index. Z = standardized values. Significance threshold ≤ 0.001. Significant estimates are bolded. Model 0: raw association. Model 1: sPMI_Z or MI_Z and age, sex, BMI, height and migration background. Model 2: sPMI_Z or MI_Z and age, sex, BMI, height, migration background and puberty score.

***STROBE CHECKLIST***

STROBE Statement—Checklist of items that should be included in reports of ***cross-sectional studies***

|  | Item No | Recommendation | Page number (main document) |
| --- | --- | --- | --- |
| **Title and abstract** | 1 | (*a*) Indicate the study’s design with a commonly used term in the title or the abstract | 3 |
|  |  | (*b*) Provide in the abstract an informative and balanced summary of what was done and what was found | 3 |
| Introduction | | |  |
| Background/rationale | 2 | Explain the scientific background and rationale for the investigation being reported | 3-5 |
| Objectives | 3 | State specific objectives, including any prespecified hypotheses | 5 |
| Methods | | |  |
| Study design | 4 | Present key elements of study design early in the paper | 5-6 |
| Setting | 5 | Describe the setting, locations, and relevant dates, including periods of recruitment, exposure, follow-up, and data collection | 5-8 |
| Participants | 6 | (*a*) Give the eligibility criteria, and the sources and methods of selection of participants | 5-8 |
| Variables | 7 | Clearly define all outcomes, exposures, predictors, potential confounders, and effect modifiers. Give diagnostic criteria, if applicable | 5-8 |
| Data sources/ measurement | 8* | For each variable of interest, give sources of data and details of methods of assessment (measurement). Describe comparability of assessment methods if there is more than one group | 6-8 |
| Bias | 9 | Describe any efforts to address potential sources of bias | 9 |
| Study size | 10 | Explain how the study size was arrived at | 5 |
| Quantitative variables | 11 | Explain how quantitative variables were handled in the analyses. If applicable, describe which groupings were chosen and why | 8-9 |
| Statistical methods | 12 | (*a*) Describe all statistical methods, including those used to control for confounding | 8-9 |
|  |  | (*b*) Describe any methods used to examine subgroups and interactions | 8-9 |
|  |  | (*c*) Explain how missing data were addressed | 5 |
|  |  | (*d*) If applicable, describe analytical methods taking account of sampling strategy | 9 |
|  |  | (*e*) Describe any sensitivity analyses | 9 |
| Results | | |  |
| Participants | 13* | (a) Report numbers of individuals at each stage of study—eg numbers potentially eligible, examined for eligibility, confirmed eligible, included in the study, completing follow-up, and analysed | 5 |
|  |  | (b) Give reasons for non-participation at each stage | 5 |
|  |  | (c) Consider use of a flow diagram | n/a  complete cases were used |
| Descriptive data | 14* | (a) Give characteristics of study participants (eg demographic, clinical, social) and information on exposures and potential confounders | 9-10 and  table 1 |
|  |  | (b) Indicate number of participants with missing data for each variable of interest | n/a  complete cases were used |
| Outcome data | 15* | Report numbers of outcome events or summary measures | 10 and table 1 |
| Main results | 16 | (*a*) Give unadjusted estimates and, if applicable, confounder-adjusted estimates and their precision (eg, 95% confidence interval). Make clear which confounders were adjusted for and why they were included | 10-11 and tables 2 and 3 |
|  |  | (*b*) Report category boundaries when continuous variables were categorized | 7-8 |
|  |  | (*c*) If relevant, consider translating estimates of relative risk into absolute risk for a meaningful time period | n/a  exposure and outcome were standardized and effect sizes relate to standard deviation change |
| Other analyses | 17 | Report other analyses done—eg analyses of subgroups and interactions, and sensitivity analyses | 10 and tables S1, S2 and S3 |
| Discussion | | |  |
| Key results | 18 | Summarise key results with reference to study objectives | 11 |
| Limitations | 19 | Discuss limitations of the study, taking into account sources of potential bias or imprecision. Discuss both direction and magnitude of any potential bias | 13 |
| Interpretation | 20 | Give a cautious overall interpretation of results considering objectives, limitations, multiplicity of analyses, results from similar studies, and other relevant evidence | 11-13 |
| Generalisability | 21 | Discuss the generalisability (external validity) of the study results | 12-13 |
| Other information | | |  |
| Funding | 22 | Give the source of funding and the role of the funders for the present study and, if applicable, for the original study on which the present article is based | 2 |

*Give information separately for exposed and unexposed groups.

**Note:** An Explanation and Elaboration article discusses each checklist item and gives methodological background and published examples of transparent reporting. The STROBE checklist is best used in conjunction with this article (freely available on the Web sites of PLoS Medicine at http://www.plosmedicine.org/, Annals of Internal Medicine at http://www.annals.org/, and Epidemiology at http://www.epidem.com/). Information on the STROBE Initiative is available at www.strobe-statement.org.
